# Insight into the Structure Evolution and Performance Optimization of Bi_0.5_Na_0.5_TiO_3_-Based Ceramics for Energy Storage Application

**DOI:** 10.3390/ma18081801

**Published:** 2025-04-15

**Authors:** Qian Wang, Lin Zhang, Rui Li, Hui Yang, Chuanhui Wang, Zhao Xiong, Chunwu Liu

**Affiliations:** 1 School of Physics and Mechanical & Electronical Engineering, Institute for Functional Materials, Hubei University of Education, Wuhan 430205, China; wangqian@hue.edu.cn (Q.W.); lirui@hue.edu.cn (R.L.); hyang@hue.edu.cn (H.Y.); 18271558809@163.com (Z.X.); 13238593107@163.com (C.L.); 2 Institute of Materials Research and Engineering, Hubei Engineering Technology Research Center of Environmental Purification Materials, Hubei University of Education, Wuhan 430205, China

**Keywords:** energy storage, ion doping, domain structure, PNRs, remnant polarization

## Abstract

The excellent temperature stability and high saturation polarization of Bi_0.5_Na_0.5_TiO_3_ (BNT) make it a promising candidate for energy storage capacitors. However, its disadvantages, such as low breakdown strength, high remnant polarization, and a complex sintering process, limit its further development. To address this, (1 − *x*) Bi_0.5_Na_0.5_TiO_3_−x Sr(Mg_1/3_Nb_2/3_)O_3_ ceramics were fabricated, where ion doping was employed to modify the domain structure, reduce the grain size, and improve the energy storage performance. With the increase in dopant concentration, the evolution from long-range-ordered ferroelectric micro-domains into short-range-ordered randomly oriented polar nanoregions (PNRs) was revealed, as demonstrated by XRD and Raman spectroscopy. This resulted in a diffuse phase transition peak and a significant reduction in remnant polarization. However, the saturation polarization also decreased. Finally, the optimal energy storage performance was achieved at a medium dopant concentration (*x* = 0.10), accompanied by reduced grain size and a dense microstructure. This composition exhibited a discharged energy density of 1.64 J/cm^3^ at a low electric field of 150 kV/cm, representing a notable improvement over pure BNT, which showed a highly lossy P-E loop and a discharged energy density of only 0.14 J/cm^3^ at the same electric field.

## 1. Introduction

Energy storage, as an intermediate unit between energy generation and consumption, is regarded as one of the key supporting technologies for the “energy revolution” [1]. Dielectric capacitors, which belong to short-time, high-frequency energy storage technology, are widely used in applications requiring the rapid release of large amounts of energy, such as pulse power technology. This technology has great significance in fields like controlled nuclear fusion ignition, pulse lasers, electromagnetic rail guns, and other national defense technologies [2]. Among commercial ceramic dielectrics, bismuth sodium titanate (Bi_0.5_Na_0.5_TiO_3_, abbreviated as BNT)-based ceramics and thin films are considered to be more promising candidates than BaTiO_3_ due to the characteristics of higher saturation polarization (43 μC/cm^2^) and excellent high-temperature stability [3,4]. However, the high remnant polarization (P_r_), low breakdown strength (BDS), and complex sintering process of this material system limit the achievement of higher energy densities [5,6,7].

In recent years, several attempts have been made to address these limitations, including ion doping at A/B sites [8,9,10,11], solid solution construction [12,13], oxide addition [14,15,16,17], chemical preparation of powders [18], and sintering process control of ceramics [19,20,21,22]. These approaches aim to reduce the P_r_ and enhance the BDS in BNT-based ceramics, ultimately achieving the synergistic optimization of energy storage performance. While the aforementioned methods primarily concern the preparation process of BNT-based ceramics, the macroscopic electrical properties of a material are often a reflection of its microscopic structure. Accordingly, research efforts focused on the structural engineering of BNT-based ceramics are more significant, both for optimizing performance and exploring the underlying mechanisms of this material system.

Due to the traditional solid-state reaction method, dielectric ceramics usually possess complex and multi-scale structures, including ion vacancies, defect dipoles, nanodomains, grains, grain boundaries, and even core–shell structures, sandwich structures, and multilayer structures [23]. Controlling a single structure can lead to the synergistic optimization of key parameters of energy storage performance. For example, modifying defect dipoles can inhibit the migration of oxygen vacancies, thereby reducing the leakage current and enhancing the BDS of BNT-based ceramics [24,25]. Additionally, the formation of defect dipoles can create an additional defect dipole polarization (P_D_), which acts as an internal field to switch the domain back to its original state after removing the external electric field, resulting in reduced P_r_ and a pinched P-E loop [26,27]. Consequently, the increased BDS, together with reduced P_r_ through the construction of defect dipoles, results in notably improved energy storage densities and efficiencies. However, greater breakthroughs can often be achieved through multi-scale structure modification. For instance, Wang et al. designed the Na_0.5_Bi_0.5_TiO_3_-Sr_0.7_Bi_0.2_TiO_3_-Ba(Mg_1/3_Ta_2/3_)O_3_ (BNST-BMT) system based on a dual optimization strategy. The introduction of Sr_0.7_Bi_0.2_TiO_3_ induced the formation of nanodomains in ceramics, leading to slim P-E curves, while the further introduction of Mg/Ta ions reduced the grain size and increased the bandgap width, resulting in significantly enhanced BDS. Ultimately, an ultrahigh energy density of 8.58 J/cm^3^ was obtained simultaneously under 565 kV/cm for the BNST-0.08BMT ceramic [28].

Based on this, BNT-based ceramic was selected as the research object in this work, and a multi-scale structure modification strategy was employed to enhance the energy storage performance. To be specific, Sr^2+^ and (Mg_1/3_Nb_2/3_)^4+^ were introduced into the BNT lattice to replace (Bi_0.5_Na_0.5_)^2+^ and Ti^4+^, respectively. Sr^2+^ and (Mg_1/3_Nb_2/3_)^4+^, with larger ionic radii and different valence states compared to the host lattice ions, are expected to induce lattice distortion, generate local random fields, and form polar nanoregions (PNRs), while also inhibiting grain growth. These effects will be carefully checked by XRD, Raman spectroscopy, and SEM. The structure evolution mentioned above may lead to reduced dielectric loss, leakage current and P_r_, while enhancing BDS, energy storage densities, and efficiencies. These outcomes will be verified through dielectric temperature spectra and P-E loops. Finally, the optimized structure and energy storage performance will be achieved in this work, and the relation between the structure evolution and the electrical properties will be established.

## 2. Experimental Procedure

### 2.1. Samples Preparation

In this work, (1 − *x*) Bi_0.5_Na_0.5_TiO_3_-*x* Sr(Mg_1/3_Nb_2/3_)O_3_ (abbreviated as (1 − *x*) BNT-*x* SMN, where *x* = 0, 0.05, 0.10, 0.20) ceramics were synthesized through traditional solid-state reaction method. High-purity commercial carbonate and oxide powders, including Na_2_CO_3_ (99.8%, Aladdin), SrCO_3_ (99.0%, Aladdin), Bi_2_O_3_ (99.0%, Aladdin), TiO_2_ (99.0%, Aladdin), MgO (99.0%, Aladdin), and Nb_2_O_5_ (99.0%, Aladdin), were weighted according to the stoichiometric ratio. The powder mixture was ball-milled with zirconium media for 24 h, using a planetary mill (MITR-QMQX-1L, MITR, Changsha, China) at a rotational speed of 240 rpm. Slurry was then dried and calcined at 850 °C for 4 h to synthesize the primary perovskite crystalline phase. Subsequently, the calcined powders were re-milled, granulated with 5 wt% polyvinyl alcohol (PVA) binder, and uniaxially pressed into green pellets with a diameter of 12 mm and a thickness of 1.5 mm. Finally, the green compacts were sintered at 1140 °C for 3 h in air, resulting in dense ceramic pellets with strong mechanical strength, being qualified for subsequent structural characterization and property evaluation.

### 2.2. Structure and Properties Characterization

The phase structures of the ceramics were examined by X-ray diffractometer (XRD) (X’Pert PRO, PANalytical, Almelo, The Netherlands) with Cu K_α1_ radiation (λ = 1.5406 Å); the working voltage is 40 kV and the current is 40 mA. The microstructures, including grain size, pore distribution, and density of ceramic samples, were observed via a field-emission scanning electron microscope (FE-SEM) (TESCAN MIRA LMS, TESCAN, Brno, Czech Republic). Specifically, the ceramic samples were polished and thermally etched at 1080 °C for 20 min to fully expose the grains, facilitating the observation of the cross-sectional microstructure. Additionally, the elemental distribution was examined by EDS mapping. To further study the local structure, including bonds and lattice, Raman spectroscopy (DXR3, Thermo Fisher Scientific, Waltham, MA, USA) with laser of 532 nm was utilized, and in order to ensure the accuracy of the test, the exposure times of the sample and background are set to be 40 times, and the entire signal acquisition time is up to 15 min.

The dielectric properties were tested on silver-plated ceramics with a precision LCR meter (E4980A, Aglient, Santa Clara, CA, USA) over a temperature range of 25–400 °C. P-E loops were measured on silver-plated ceramics polished to a thickness of 0.3 mm, using a ferroelectric workstation (PKCPE1701, PolyK, Philipsburg, PA, USA) at a frequency of 10 Hz and a temperature of 25 °C.

## 3. Results and Discussion

### 3.1. Structure of (1 − x) BNT-x SMN Ceramics

**Figure 1:**
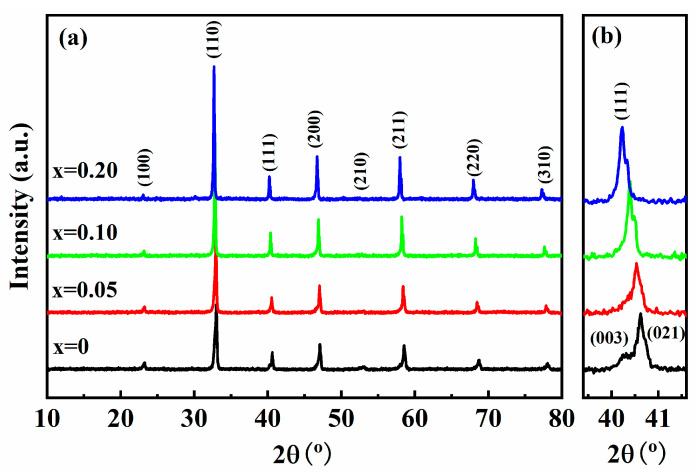
XRD patterns of the (1 − *x*) BNT-*x* SMN ceramics in the 2θ range of (**a**) 10–80° and (**b**) 39–42°.

Figure 1 shows the XRD patterns of the (1 − *x*) BNT-*x* SMN ceramics, which exhibit a typical perovskite structure with no detectable secondary phase. All ions of SMN have entered the BNT lattice and formed a uniform solid solution. However, a closer examination of the XRD patterns, as shown in Figure 1b, reveals further insights. Firstly, the diffraction peaks shift to lower angles with increasing *x*, indicating a gradual increase in the lattice parameter. This phenomenon can be attributed to ion doping. To be specific, Sr^2+^ was designed to replace (Bi_0.5_Na_0.5_)^2+^ at the A-site in this work, while (Mg_1/3_Nb_2/3_)^4+^ substitutes Ti^4+^ at the B-site. The ionic radii of Sr^2+^, Mg^2+^, and Nb^5+^ are 1.44 Å, 0.72 Å, and 0.64 Å, respectively, as reported in the literature [29], which are larger than those of Bi^3+^, Na^+^, and Ti^4+^ (1.38 Å, 1.39 Å, and 0.604 Å, respectively); thus, the incorporation of dopants with larger ionic radii leads to lattice expansion.

Additionally, for *x* = 0, the splitting of the (003)/(021) peaks at around 41° signifies the existence of the rhombohedral phase, which is consistent with previously reported on pure BNT [30]. With increasing SMN content, the split peaks gradually merge into a single peak of (111), indicating a phase evolution from rhombohedral to pseudocubic. Typically, the pseudocubic phase suggests the coexistence of rhombohedral (R3c) and tetragonal (P4bm) phases in the ceramic samples, accompanied by the formation of polar nanoregions (PNRs). This structural evolution directly correlates with the attenuation of macroscopic ferroelectricity.

**Figure 2:**
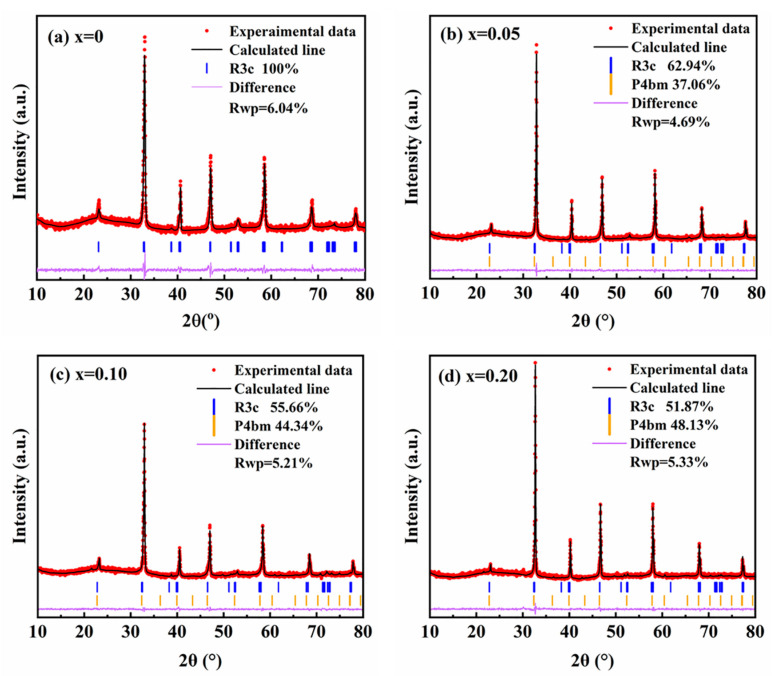
The Rietveld refinement of XRD data for the (1 − *x*) BNT-*x* SMN ceramics.

To further verify the fractions of R3c and P4bm phase, Rietveld refinement was performed on the XRD patterns, and the refinement results are shown in Figure 2. It can be seen that pure BNT exclusively comprises the R3c phase, whereas SMN-doped BNT exhibit the coexistence of R3c and P4bm phase. Notably, the R3c phase fraction decreases with increasing SMN content. Furthermore, Rietveld-refined lattice parameters reveal a gradual expansion of the unit cell (Table 1), a trend consistent with the XRD peak shifting observed in Figure 1.

The phase evolution with increasing SMN content can be reasonably explained by the local charge imbalance and ionic radius difference in the dopants, which induce the local random fields. Once the local random fields are generated, the long-range-ordered ferroelectric micro-domains are disrupted, resulting in the formation of mixed PNRs with R3c and P4bm phases. These PNRs play a key role in modulating the dielectric and ferroelectric properties, finally contributing to the high performance of energy storage ceramics.

**Figure 3:**
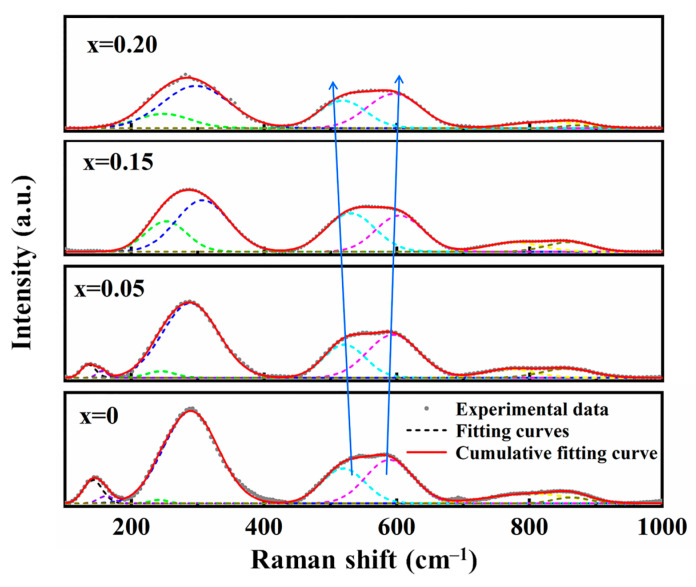
Raman spectra of the (1 − *x*) BNT-*x* SMN ceramics.

To further study the effect of ion doping on the local ionic configurations of BNT ceramics, Raman spectroscopy was employed, and the Gaussian functions were used for spectral deconvolution using Origin software (Origin 2018 64Bit). The results are shown in Figure 3. As reported in most research works about BNT-based dielectrics, four groups of vibration modes are detected in the wavenumber ranges of 100–200 cm^−1^, 200–400 cm^−1^, 440–690 cm^−1^, and above 690 cm^−1^, corresponding to the vibration of A-site ions, the B-O bond, the titanium–oxygen octahedron (TiO_6_), and the overlapping of A1 and E bands, respectively.

With increasing SMN content, the modes related to the vibrations of A-site ions and B-O bond are broadened and depressed, indicating an increased degree of disorder at both the A-site and B-site due to the ion doping. A similar phenomenon is observed for the modes associated with the vibration of TiO_6_ octahedron. Specifically, these modes are broadened with increasing SMN amount, suggesting a distortion of the TiO_6_ octahedron and a reduction in the size of PNRs. The Raman spectra analysis is in good agreement with the XRD results, indicating that the local charge mismatch and atomic spacing expansion caused by ion doping lead to lattice distortion. This lattice distortion generates local random fields, promotes the formation of PNRs, and ultimately results in broadened dielectric peaks and pinched P-E loops.

**Figure 4:**
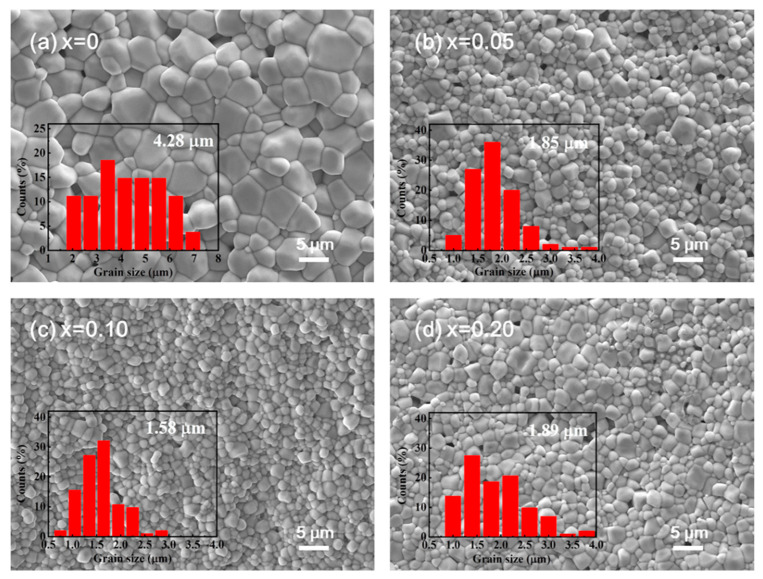
SEM images on the thermally etched cross-section of the (1 − *x*) BNT-*x* SMN ceramics (the magnification is 2000). The insets show the grain size distribution.

**Figure 5:**
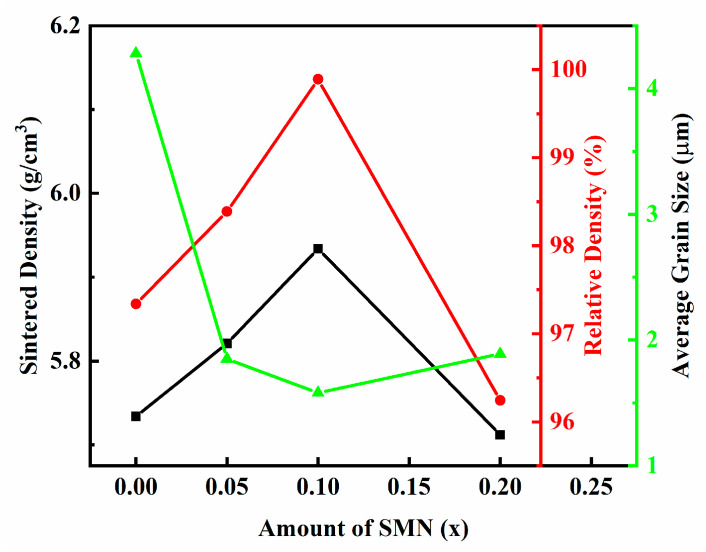
The sintered densities, relative densities, and average grain size of the (1 − *x*) BNT-*x* SMN ceramics.

Figure 4 gives the SEM images of the thermally etched cross-section for (1 − *x*) BNT-*x* SMN ceramics, and Figure 5 gives the sintered densities, relative densities and average grain size of all the samples, revealing spheroidal grains and a nearly pore-free morphology. This indicates that the sintering procedure (1140 °C × 3 h) is suitable for grain growth and pore removal. However, grain growth is notably inhibited by ion doping. The average grain size of pure BNT is 4.28 μm, while it decreases to 1.85 μm for 0.95 BNT-0.05 SMN, as given in Figure 4 and Figure 5. The reduction in average grain size with increasing dopant content can be explained by two factors: (1) the doped ions, with their different radii, induce lattice deformation and rotation, increasing lattice strain energy, and hindering grain boundary activity [31]; and (2) the reduction in BNT content lowers the concentration of volatile Na^+^ and Bi^3+^, thereby reducing oxygen vacancy concentration and inhibiting mass transfer during sintering.

In general, the reduced average grain size is favorable for suppressing the dielectric loss and leakage current, while enhancing the breakdown strength. This phenomenon can be explained by analyzing the expansion of electrical trees under the external electric field. It is well-known that ceramics are not electrically homogeneous and generally contain grains with lower insulating properties and grain boundaries with higher insulating properties. Under the external electric field, breakdown initially occurs locally within the grains, forming micro-cracks. As the electric field increases, these micro-cracks begin to expand, forming the electrical tree. However, the expansion of electrical trees can be prevented when encountering grain boundaries due to the much higher breakdown energy of the grain boundary. Therefore, the reduction in grain size leads to the increasing of the grain boundary density, which shows a stronger inhibition effect on the expansion of electrical trees. As a result, dielectric loss and leakage current are significantly reduced, while the breakdown strength of the ceramic sample is usually enhanced.

Nevertheless, grain refinement and uniform grain size distribution are achieved only with an appropriate amount of dopants (*x* = 0.05 and 0.10), as can be seen in the insets of Figure 4. The grain size of the *x* = 0 sample is distributed in a relatively wide range of 2–7 μm, compared with that of the *x* = 0.05 (1–4 μm) and 0.10 (1–3 μm) sample, indicating a more uniform grain morphology after the addition of SMN. Excessive doping (*x* = 0.20) leads to microstructural deterioration, such as slightly increased grain size and non-uniform grain size distribution. Generally, in cases of excessive doping, ions accumulate at the grain boundaries rather than entering the lattice, inhibiting the movement of grain boundaries during the sintering process. This results in abnormal grain growth, which typically has undesirable effects on the electrical properties of ceramics, including increased leakage current and reduced breakdown strength.

**Figure 6:**
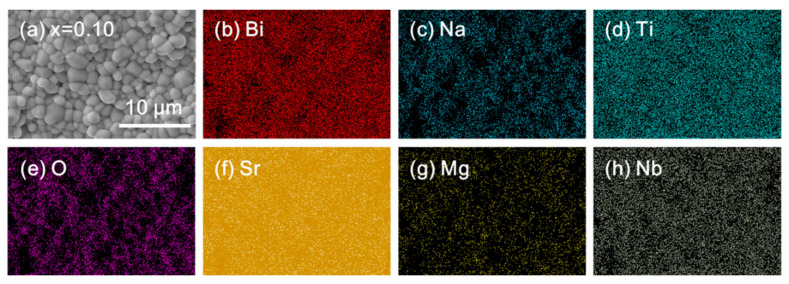
(**a**) Enlarged SEM morphology (the magnification is 5000) and (**b**–**h**) EDS mapping images of 0.9 BNT-0.1 SMN ceramics.

Additionally, all elements are uniformly distributed in ceramics with moderate doping, such as *x* = 0.10, as demonstrated in Figure 6, indicating that Sr^2+^, Mg^2+^, and Nb^5+^ have successfully entered the lattice and replaced the ions at the A/B sites, consistent with the XRD results.

### 3.2. Dielectric Properties of (1 − x) BNT-x SMN Ceramics

**Figure 7:**
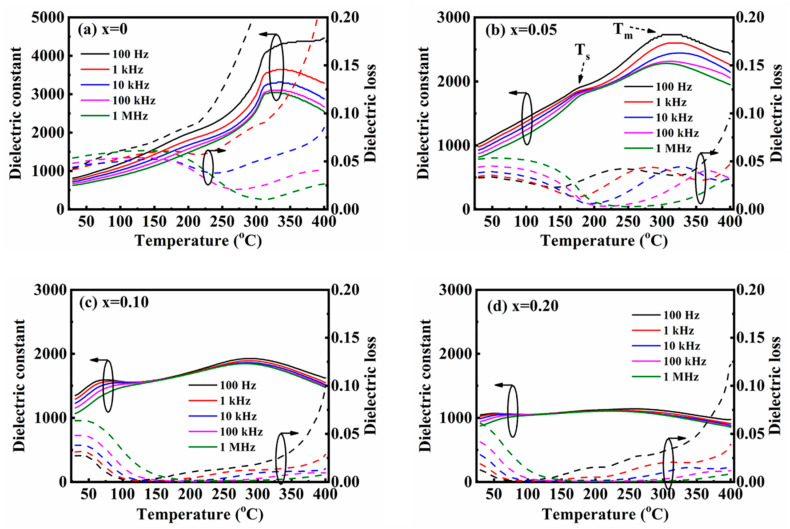
Dielectric temperature spectra of the (1 − *x*) BNT-*x* SMN ceramics.

The dielectric temperature spectra of (1 − *x*) BNT-*x* SMN ceramics were measured at 100 Hz, 1 kHz, 10 kHz, 100 kHz, and 1 MHz, as shown in Figure 7. Obvious frequency dispersion was observed, indicating the relaxor characteristics of this material system. Two dielectric anomalies, denoted as Ts and Tm and marked with dotted arrows, were observed for all ceramic samples. Typically, the thermal evolution of mixed PNRs with rhombohedral (R3c) and tetragonal (P4bm) phases is responsible for the appearance of Ts, while the transformation from R3c to P4bm phase and the thermal evolution of P4bm PNRs result in the existence of Tm.

**Figure 8:**
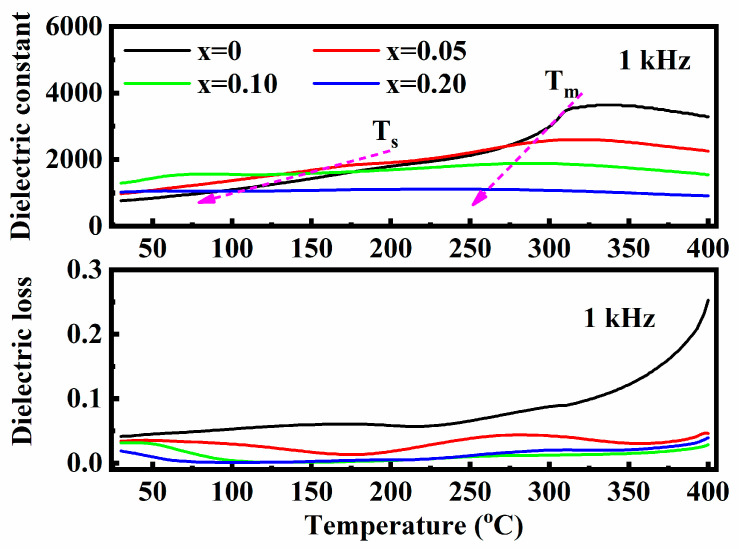
Dielectric temperature spectra of the (1 − *x*) BNT-*x* SMN ceramics at 1 kHz.

With increasing SMN content, both the peaks of Ts and Tm were suppressed and shifted to a lower temperature. However, the dielectric constant plateaus over the temperature range of Ts and Tm were broadened. Actually, these dielectric constant plateaus are ascribed to the mutual evolution of R3c and P4bm PNRs. Thus, the broadened and depressed plateaus can be reasonably explained by the introduction of Sr^2+^, Mg^2+^, and Nb^5+^, which have larger ionic radii and different valence states compared to Bi^3+^, Na^+^, and Ti^4+^. As comprehensively studied in the structure of the ceramics, ion doping generates local random fields, disrupting the long-range-ordered ferroelectric micro-domains and forming short-range-ordered randomly oriented mixed PNRs with R3c phase and P4bm phase. This leads to a dispersive phase transition from a non-ergodic relaxor state to an ergodic relaxor state, resulting in broadened and depressed dielectric plateaus, accompanied by notably reduced dielectric loss, as shown in Figure 8. This phenomenon has also been reported in other BNT-based ceramics modulated by ionic doping. Moreover, the broadened dielectric plateau typically leads to improved temperature stability for energy storage properties, which is crucial in the field of high-temperature energy storage.

**Figure 9:**
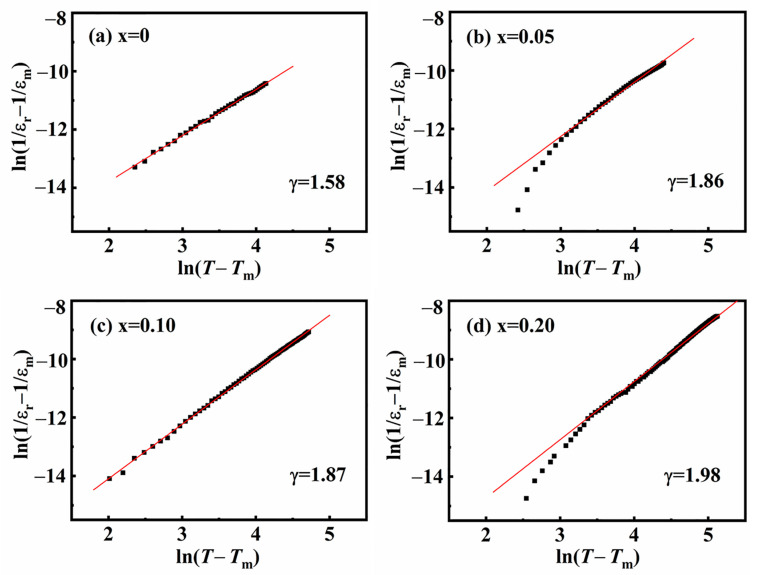
Curie–Weiss fitting of the dielectric temperature spectra of (1 − *x*) BNT-*x* SMN ceramics at 1 kHz.

To further illustrate the effect of ion doping on the dielectric properties of BNT ceramics, Curie–Weiss fitting of the dielectric temperature spectra was performed, guided by the Curie–Weiss law: $\frac{1}{\varepsilon_{r}}-\frac{1}{\varepsilon_{m}}=\frac{{\left(T-T_{m}\right)}^{\gamma }}{C}$, where the value of *γ* reflects the degree of diffuseness. Typically, *γ* = 1 indicates that the material is a typical ferroelectric, while *γ* = 2 suggests that the material is an ideal relaxor. The fitting results, as presented in Figure 9, show that the value of *γ* gradually increases with increasing SMN content, indicating an enhancement in the degree of relaxor behavior, implying the formation of more PNRs. The fitting result is consistent with the above analysis of XRD and Raman spectra.

### 3.3. Ferroelectric Properties of (1 − x) BNT-x SMN Ceramics

**Figure 10:**
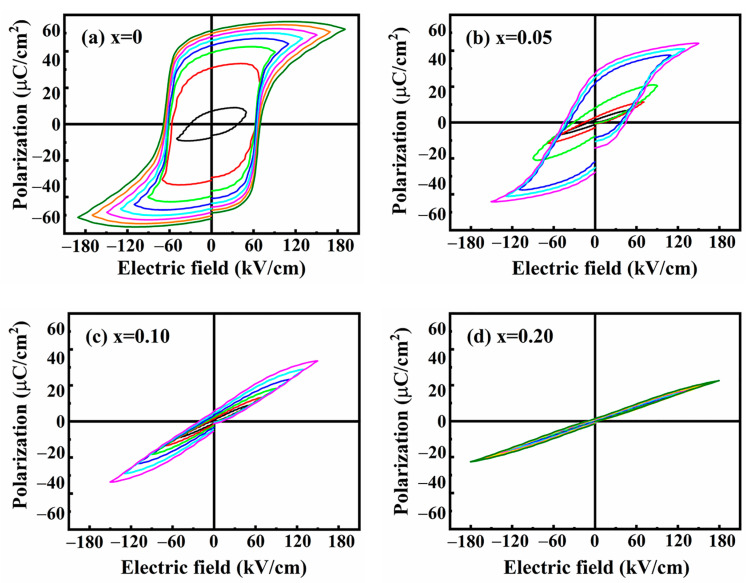
P-E loops of the (1 − *x*) BNT-*x* SMN ceramics measured at room temperature.

The P-E loops of (1 − *x*) BNT-*x* SMN ceramics were measured and are depicted in Figure 10, revealing the significant effect of ion doping on the domain structure of the ceramic samples. As the SMN content increased, the P-E loops become slimmer, implying a transition from long-range-ordered ferroelectric micro-domains to short-range-ordered randomly oriented PNRs. As demonstrated in the structural analysis above, the main crystal phase of pure BNT ceramics is the rhombohedra ferroelectric phase, which exhibits a slow response to an external electric field, resulting in a highly lossy P-E loop. With the increase in SMN content, PNRs with rhombohedra and tetragonal phase were induced. These isolated PNRs in the ceramics exhibit weakly coupled interactions and are highly sensitive to the switch of electric fields. They can rapidly assemble into a long-range ferroelectric structure when an external electric field is applied, leading to a high saturation polarization (P_max_). On the contrary, when the electric field is removed, the long-range ferroelectric micro-domains quickly collapse back into the original PNRs, resulting in low remnant polarization (P_r_) and coercive electric field (E_c_), as shown in Figure 10.

This phase evolution is desirable for the reduction of P_r_ and the enhancement of energy storage efficiency (η). However, the suppression of P_max_ is inevitable, which is unfavorable for the optimization of energy storage density. Consequently, the optimal energy storage performance can be achieved through moderate ion doping.

**Figure 11:**
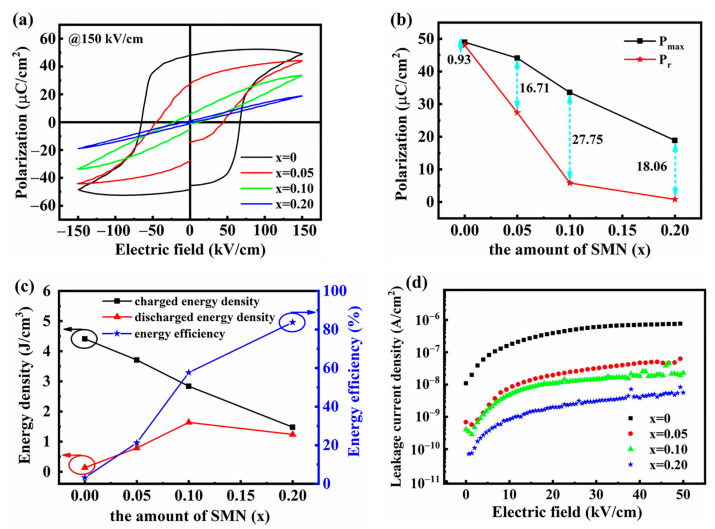
(**a**) P-E loops of the (1 − *x*) BNT-*x* SMN ceramics measured under the same electric field of 150 kV/cm, (**b**) P_max_ and P_r_ as a function of SMN content, (**c**) energy storage parameters as a function of SMN content, (**d**) leakage current density of the (1 − *x*) BNT-*x* SMN ceramics.

To further confirm the optimal composition for energy storage, the P-E loops of (1 − *x*) BNT-*x* SMN ceramics were measured under the same electric field of 150 kV/mm, as given in Figure 11a. The reduction in both P_max_ and P_r_ was observed, and their evolution trends with increasing SMN content are illustrated in Figure 11b. As the SMN content increased, the values of P_r_ decreased more sharply than those of P_max_, resulting in a widened gap between P_max_ and P_r_. This gap reached its peak at the composition of *x* = 0.10. Figure 11c gives the energy storage parameters of (1 − *x*) BNT-*x* SMN ceramic samples. With increasing SMN content, the charged energy density decreased, while the energy storage efficiency increased. Notably, the discharged energy density initially increased and then slightly decreased, reaching a peak value of 1.64 J/cm^3^ (at 150 kV/mm) for the composition of *x* = 0.10. This represents a significant improvement over pure BNT, which exhibited a highly lossy P-E loop and a much lower discharged energy density of 0.14 J/cm^3^ (at 150 kV/mm). Additionally, the leakage current densities for (1 − *x*) BNT-*x* SMN ceramics were measured and are shown in Figure 11d. As predicted, the leakage current densities decreased with increasing SMN concentration due to the synergistic effect of refined grain size and enhanced relaxor characteristic, consistent with the above structural analysis.

## 4. Conclusions

(1 − *x*) BNT-*x* SMN solid solution ceramics were fabricated via the solid-state reaction method in this work, aiming to moderate the multi-scale structure and enhance the energy storage performance. A transition from long-range-ordered ferroelectric micro-domains to short-range-ordered randomly oriented PNRs was confirmed by the analysis of XRD and Raman spectra, meanwhile the grain growth was effectively restrained. The modification of domain structure and microstructure resulted in broadened and depressed dielectric plateaus, reduced remnant polarization, and suppressed leakage current densities. Finally, the highest discharged energy density of 1.64 J/cm^3^ (at 150 kV/mm) was achieved for the composition with x = 0.10, representing a significant improvement over pure BNT, which exhibited a highly lossy P-E loop and a much lower discharged energy density of 0.14 J/cm^3^ (at 150 kV/mm). This work provides insight into the structure evolution and performance optimization of Bi_0.5_Na_0.5_TiO_3_-based ceramics, demonstrating that multi-scale structure engineering can enhance the electrical properties of ceramics, thereby providing both experimental and theoretical support for related research.

## Figures and Tables

**Table 1:** Refined lattice parameters of the (1 − *x*) BNT-*x* SMN ceramics.

	Space Group	Weight Fraction (%)	Lattice Parameters	V (Å^3^)	Rwp (%)
*x* = 0	R3c	100.00	a = b=5.5092, c = 13.4314	353.0471	6.04
			α = β = 90°, γ = 120°		
*x* = 0.05	R3c	62.94	a = b = 5.5145, c = 13.5018	355.5807	4.89
			α = β = 90°, γ = 120°		
	P4bm	37.06	a = b = 5.5427, c = 3.9046	119.9563	
			α = β = γ = 90°		
*x* = 0.10	R3c	55.66	a = b = 5.5033, c = 13.5063	354.2575	5.21
			α = β = 90°, γ = 120°		
	P4bm	44.34	a = b = 5.5027, c = 4.1839	126.6869	
			α = β = γ = 90°		
*x* = 0.20	R3c	51.87	a = b = 5.5347, c = 13.5676	359.9321	5.33
			α = β = 90°, γ = 120°		
	P4bm	48.13	a = b = 5.5387, c = 4.1865	128.4293	
			α = β = γ = 90°		

## Data Availability

The original contributions presented in this study are included in the article. Further inquiries can be directed to the corresponding authors.
